# Improved Malaria Case Management through the Implementation of a Health Facility-Based Sentinel Site Surveillance System in Uganda

**DOI:** 10.1371/journal.pone.0016316

**Published:** 2011-01-19

**Authors:** Asadu Sserwanga, Jamal C. Harris, Ruth Kigozi, Manoj Menon, Hasifa Bukirwa, Anne Gasasira, Stella Kakeeto, Fred Kizito, Ebony Quinto, Denis Rubahika, Sussann Nasr, Scott Filler, Moses R. Kamya, Grant Dorsey

**Affiliations:** 1 Uganda Malaria Surveillance Project, Kampala, Uganda; 2 Department of Pediatrics, University of California San Francisco, San Francisco, California, United States of America; 3 Centers for Disease Control and Prevention, Atlanta, Georgia, United States of America; 4 Uganda Ministry of Health, Kampala, Uganda; 5 Department of Medicine, Makerere University School of Medicine, Kampala, Uganda; 6 Department of Medicine, University of California San Francisco, San Francisco, California, United States of America; Kenya Medical Research Institute - Wellcome Trust Research Programme, Kenya

## Abstract

**Background:**

Heath facility-based sentinel site surveillance has been proposed as a means of monitoring trends in malaria morbidity but may also provide an opportunity to improve malaria case management. Here we described the impact of a sentinel site malaria surveillance system on promoting laboratory testing and rational antimalarial drug use.

**Methodology/Principal Findings:**

Sentinel site malaria surveillance was established at six health facilities in Uganda between September 2006 and January 2007. Data were collected from all patients presenting to the outpatient departments including demographics, laboratory results, diagnoses, and treatments prescribed. Between the start of surveillance and March 2010, a total 424,701 patients were seen of which 229,375 (54%) were suspected of having malaria. Comparing the first three months with the last three months of surveillance, the proportion of patients with suspected malaria who underwent diagnostic testing increased from 39% to 97% (p<0.001). The proportion of patients with an appropriate decision to prescribe antimalarial therapy (positive test result prescribed, negative test result not prescribed) increased from 64% to 95% (p<0.001). The proportion of patients appropriately prescribed antimalarial therapy who were prescribed the recommended first-line regimen artemether-lumefantrine increased from 48% to 69% (p<0.001).

**Conclusions/Significance:**

The establishment of a sentinel site malaria surveillance system in Uganda achieved almost universal utilization of diagnostic testing in patients with suspected malaria and appropriate decisions to prescribed antimalarial based on test results. Less success was achieved in promoting prescribing practice for the recommended first-line therapy. This system could provide a model for improving malaria case management in other health facilities in Africa.

## Introduction

Malaria surveillance is essential to guide program planning and management, inform governments and donors on progress towards malaria control, and assist with advocacy. Surveillance also provides the basis for the design, refinement and resource allocation of control programs [1], [2]. Most malaria control programs in Africa rely on routinely collected health facility-based data for surveillance needs as part of a country's Health Management Information Services (HMIS). The methods used to collect data at health facilities vary widely and are highly subject to bias, as there are many factors that influence whether a patient with malaria will be captured by this system [3]. In addition, health facility data may be inaccurate due to lack of reporting and/or the absence of laboratory confirmation of diagnoses. Indeed, national reports on trends in malaria cases from most countries in Africa simply reflect the number of unconfirmed cases captured through the HMIS system [3]. The slide positivity rate (SPR), defined as the number of laboratory-confirmed malaria cases per 100 suspected cases examined, provides an alternative method for estimating temporal changes in malaria morbidity. The SPR gains accuracy in considering only laboratory confirmed cases of malaria, and it can provide a rapid and inexpensive means of assessing the burden of malaria in a population utilizing health care facilities. However, the SPR is subject to bias and is dependent on a number of factors influencing whether a suspected case undergoes laboratory testing and test results are accurately measured and reported. Decreases in the SPR have been cited as evidence for successful malaria control interventions in Africa [4], [5].

Unfortunately most health facilities in Africa currently lack the capacity to generate accurate data on malaria cases based on laboratory confirmation [3]. Sentinel site surveillance has been proposed as a practical means of improving the quality of malaria surveillance data in Africa at selected health facilities [6]. Sentinel sites are health facilities from a limited number of geographically defined areas selected to produce high quality malaria surveillance data based on laboratory confirmed cases. In addition to improving the accuracy of malaria surveillance data, laboratory confirmed diagnosis may promote the rational use of antimalarial therapy and improve patient care. Indeed, recently published guidelines from the World Health Organization (WHO) now recommend a laboratory confirmation of diagnosis in all patients suspected of having malaria before treating [7]. This updated approach to malaria case management follows the widespread adoption of highly effective but relatively expensive artemisinin-based combination therapy (ACT), signifying a need for restricted and better targeted treatment of malaria.

In 2006, the Uganda Malaria Surveillance Project (UMSP) in collaboration with the Ugandan Ministry of Health established a sentinel site malaria surveillance system with support from the U.S. President's Malaria Initiative. Sites were purposefully selected to represent the varying epidemiology of malaria in Uganda. The primary objective of this surveillance system was to provide a means of accurately monitoring trends in malaria morbidity based on the SPR. However, establishing this system also provided an opportunity to improve the utilization of diagnostic testing and promote the rational use of antimalarial therapy. Data were previously published on the impact of a focused short-course training program on malaria case management 3–6 months following the establishment of the surveillance system [8]. Here we describe the maintenance of the UMSP sentinel site surveillance system and the longer-term impact that this had on malaria case management over 3 years following its implementation

## Methods

### Establishment of health facility-based surveillance system and study sites

**Figure 1:**
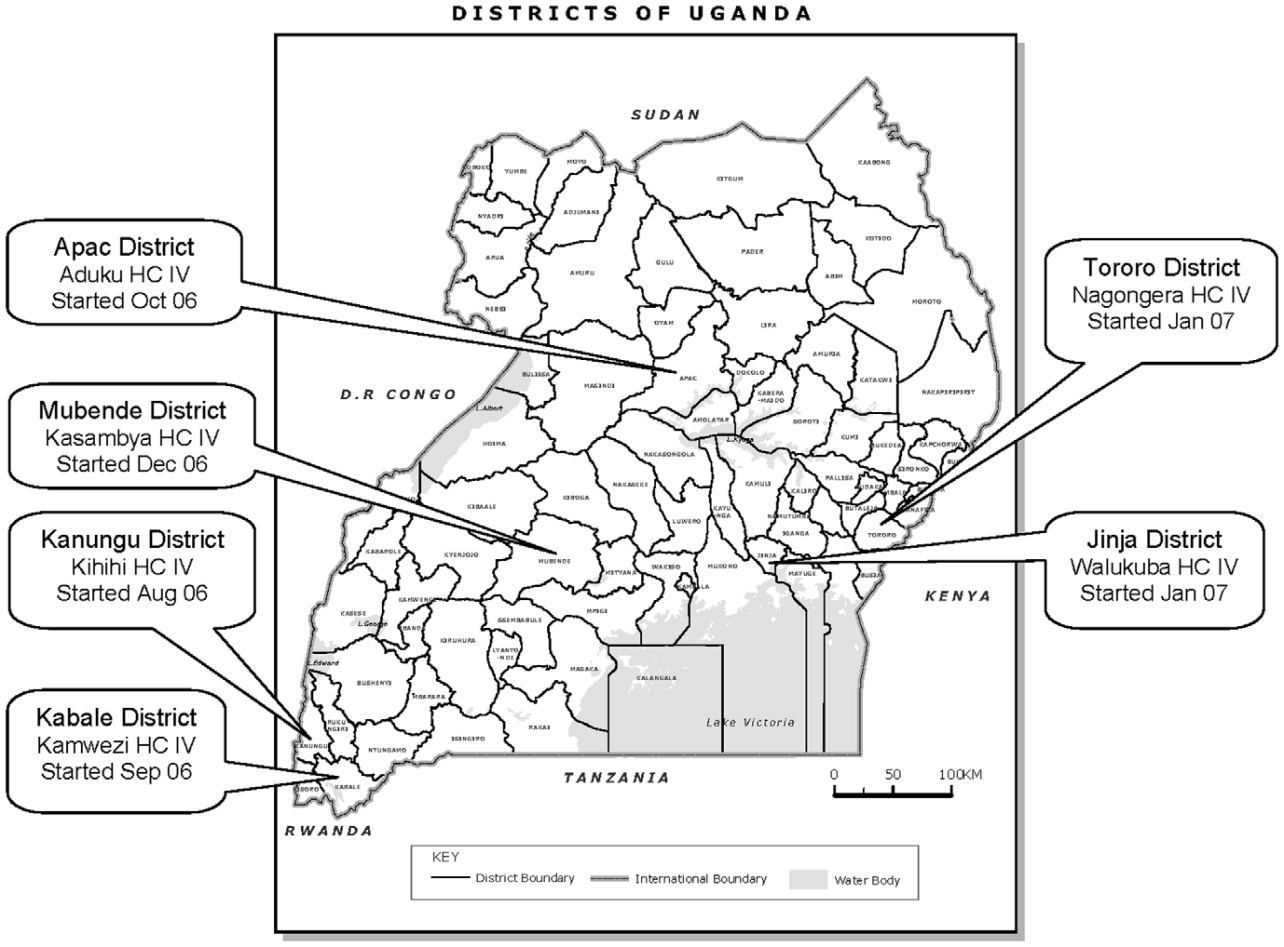
Map of the districts, government health centers, and dates of initiation for the UMSP sentinel sites.

UMSP in collaboration with the Uganda National Malaria Control Program (NMCP) established a health facility-based malaria surveillance system at six sentinel sites between September 2006 and January 2007 (Figure 1). All sentinel site facilities are government run level IV health centers that provide care free of charge, including diagnostic testing and medications. Level IV health centers generally have a catchment population of approximately 100,000 people and are staffed by one medical officer, two clinical officers, five nurses, five midwives, four nursing assistants, one dental officer, one lab technician, one lab assistant, one records officer, one health educator and one health assistant. Each site had previously been selected as part of the East African Network for Monitoring Antimalarial Treatment (EANMAT) to represent the diversity of geography and malaria transmission intensity in Uganda [9].

### Data collection and management

Methods developed to collect data were part of an iterative and collaborative process between UMSP and the Uganda HMIS. Unlike the traditional HMIS, the new data collection system provided individual-level data on all patients presenting to the outpatient department of the sentinel site facilities. Data collected included patient demographic information, basic clinical information, laboratory results, diagnoses, and treatments prescribed.

Data collection instruments evolved from written clinical and laboratory log books to a single standardized case record form (CRF) completed for each patient and characterized by tick boxes and lists of options to minimize transcription errors (Appendix S1). Data collection instruments were initially transported to a core facility in the capital city of Kampala for electronic data entry using Access (Microsoft Corporation, Redmond, WA). Over time this system advanced to where UMSP employed data officers entered the data electronically using Epi Info version 3.5.1 (Centers for Disease Control and Prevention, Atlanta, GA) at the sites and sent it remotely once a month via cellular phone technology to a core facility in Kampala for uploading to a SQL server (Microsoft Corporation, Redmond, WA). Data officers were the only direct staff supported by UMSP at the sentinel sites. A public website exists (http://umsp.muucsf.org) where standardized tables and figures can be generated to monitor trends in key indicators and monthly reports are posted.

### Training and quality assurance

Staff at the sentinel site facilities underwent a six-day training course 3–6 months following the initiation of the surveillance program to improve health workers' performance of clinical and laboratory tasks relevant to malaria case management. The curriculum and training materials were developed and delivered through the Joint Uganda Malaria Training Program (JUMP), a partnership between UMSP and the Infectious Diseases Institute (IDI) of Makerere University, the details of which have been previously published [8]. The course was team-based and targeted three categories of staff typically working in health facilities in Uganda: clinicians (medical officers, clinical officers, nurses and midwives), laboratory staff, and records clerks. The course included both didactic and practical hands-on sessions. Two follow-up support supervision visits, approximately 6 and 12 weeks after the initial training course, were conducted at the sentinel sites by JUMP team members to reinforce training messages, assess skills, and provide individual feedback.

Following the formal training program, the UMSP surveillance team consisting of clinical, laboratory, and data officers visited the sites every 1–2 months to ensure an adequate supply of CRFs and laboratory consumables and provide feedback to staff. Feedback included review of overall site performance relative to quality assurance/quality control targets. In addition, workshops were held with district leaders in collaboration with representatives of the NMCP to build support for the project. Supervision and funding of all site visits and workshops were provided by UMSP. The project did not have any influence over drug supplies or the monitoring of stock outs.

### Malaria case management indicators and statistical analysis

**Figure 2:**
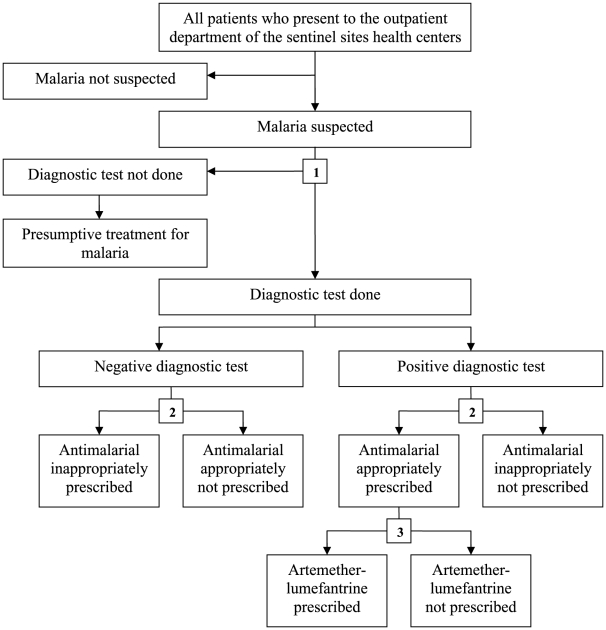
Malaria case management decision algorithm. Numbers highlight the three key indicators of malaria case management evaluated.

The primary purpose of the surveillance project was to monitor trends in malaria morbidity. However, a successful surveillance program also provides the opportunity to improve malaria case management, which was the focus of this report. Three key indicators of malaria case management were evaluated (Figure 2): 1) proportion of patients with suspected malaria with a diagnostic test done (microscopy or RDT); (suspected malaria was defined as all patients referred for malaria laboratory testing plus all patients not referred for a malaria laboratory test but given a clinical diagnosis of malaria); 2) proportion of patients with a diagnostic test done with appropriate decision to prescribe antimalarial therapy (defined as either prescribing antimalarial therapy if the result of the diagnostic test was positive or not prescribing antimalarial therapy if the result of the diagnostic test was negative); 3) proportion of patients appropriately prescribed antimalarial therapy (antimalarial prescribed with a positive diagnostic test result) who were prescribed artemether-lumefantrine (AL), the recommended 1st-line therapy for uncomplicated malaria in Uganda since 2005.

Data were included from the time surveillance began through March 2010, with the exception of May 2007 – August 2008 at one site (Kamwezi) when surveillance was interrupted due to administrative difficulties. Proportions during the first three months of surveillance were compared with the last three months (January – March 2010) using the non-parametric McNemar's test. A p-value <0.05 was considered statistically significant.

## Results

### Descriptive data

Quarterly aggregate data for all sites actively participating in the surveillance project are presented in Table 1. A total of 424,701 patients were seen at the six sites during a total of 232 months of data collection. The average number of patients seen per month ranged from 968 (Kasmbya) to 2,488 (Kamwezi), and was fairly consistent during the observation period with the exception of Kamwezi where the average number of patients seen per month increased almost 2-fold following May 2009 (1,907 vs. 3,476) due to a presumed malaria epidemic in the southwest part of the country. Overall, 229,375 patients evaluated had suspected malaria (54%); this proportion ranged from 47% (Aduku) to 64% (Kasambya). The proportion of diagnostic tests that were positive for malaria parasites was 40% and ranged from 33% (Kihihi) to 55% (Aduku). The proportion of patients seen with suspected malaria and the proportion of diagnostic tests that were positive were fairly consistent over the observation period at all of the sites with the exception of Kamwezi, where suspected malaria cases increased from 35% to 59% and the proportion of positive diagnostic tests increased from 26% to 50% following the presumed malaria epidemic in May 2009.

**Table 1:** Absolute numbers and proportions for key steps in malaria case management algorithm (all sites combined).

Time period	Number of active sites	Malaria suspected	(%)	Laboratory test done	(%)	Positive laboratory test	(%)	Appropriate decision to prescribe antimalarial	(%)	Prescribed AL	(%)
		Total patients seen		Malaria suspected		Laboratory test done		Laboratory test done		Appropriately prescribed antimalarial therapy	
Aug-Sep 06	2	2,953	(59%)	1,659	(56%)	579	(35%)	1,008	(61%)	228	(49%)
		5,038		2,953		1,659		1,659		469	
Oct-Dec 06	4	8,287	(51%)	4,417	(53%)	1,635	(37%)	3,404	(77%)	954	(72%)
		16,254		8,287		4,417		4,417		1,322	
Jan-Mar 07	6	19,384	(57%)	8,836	(46%)	3,695	(42%)	6,721	(76%)	1,880	(58%)
		23,778		19,384		8,836		8,836		3,253	
Apr-Jun 07	6	12,766	(46%)	6,616	(52%)	2,356	(36%)	5,675	(86%)	1,381	(63%)
		27,851		12,766		6,616		6,616		2,200	
Jul-Sep 07	5	11,078	(48%)	6,289	(57%)	2,876	(46%)	5,577	(89%)	1,248	(46%)
		23,138		11,078		6,289		6,289		2,737	
Oct-Dec 07	5	10,756	(49%)	6,416	(60%)	1,849	(29%)	5,765	(90%)	1,480	(84%)
		21,888		10,756		6,416		6,416		1,764	
Jan-Mar 08	5	11,212	(50%)	6,264	(56%)	2,117	(34%)	5,547	(89%)	1,788	(87%)
		22,540		11,212		6,264		6,264		2,048	
Apr-Jun 08	5	13,837	(54%)	8,448	(61%)	3,528	(42%)	7,612	(90%)	2,972	(89%)
		25,393		13,837		8,448		8,448		3,339	
Jul-Sep 08	6	12,433	(51%)	7,670	(62%)	2,841	(37%)	6,421	(84%)	2,079	(82%)
		24,527		12,433		7,670		7,670		2,542	
Oct-Dec 08	6	17,303	(49%)	12,081	(70%)	4,136	(34%)	10,277	(85%)	2,657	(68%)
		35,097		17,303		12,081		12,081		3,889	
Jan-Mar 09	6	15,038	(48%)	11,388	(76%)	3,961	(35%)	9,673	(85%)	2,258	(59%)
		31,181		15,038		11,388		11,388		3,826	
Apr-Jun 09	6	18,608	(52%)	15,125	(81%)	6,070	(40%)	13,488	(89%)	3,415	(58%)
		35,686		18,608		15,125		15,125		5,888	
Jul-Sep 09	6	21,554	(53%)	19,333	(90%)	7,549	(39%)	17,974	(93%)	4,659	(63%)
		40,309		21,554		19,333		19,333		7,349	
Oct-Dec 09	6	26,342	(64%)	24,836	(94%)	11,983	(48%)	23,413	(94%)	9,427	(80%)
		41,432		26,342		24,836		24,836		11,817	
Jan-Mar 10	6	27,824	(69%)	26,900	(97%)	12,123	(45%)	25,447	(95%)	8,182	(69%)
		40,589		27,824		26,900		26,900		11,929	
**Totals**	**-**	**229,375**	**(54%)**	**166,278**	**(72%)**	**67,298**	**(40%)**	**148,002**	**(89%)**	**44,608**	**(69%)**
		**424,701**		**229,375**		**166,278**		**166,278**		**64,372**	

### Diagnostic testing

A total of 166,278 patients underwent diagnostic testing for malaria parasites over the observation period (Table 1). Microscopy and RDTs made up 84% and 16% of diagnostic tests performed, respectively. The use of RDTs first became available in January 2009 and RDTs were only used at four of the six sites. A majority of RDT usage came from a single site (Kamwezi) where this made up 89% of diagnostic testing done starting in January 2009. At another site (Kihihi) RDTs made up 75% of diagnostic testing done between April 2009 and January 2010 when supplies were exhausted. In two other sites RDTs made up 16% (Nagongera) and 5% (Kasambya) of diagnostic testing during the brief periods they were available (May – December 2009 and November 2009 – February 2010, respectively).

**Figure 3:**
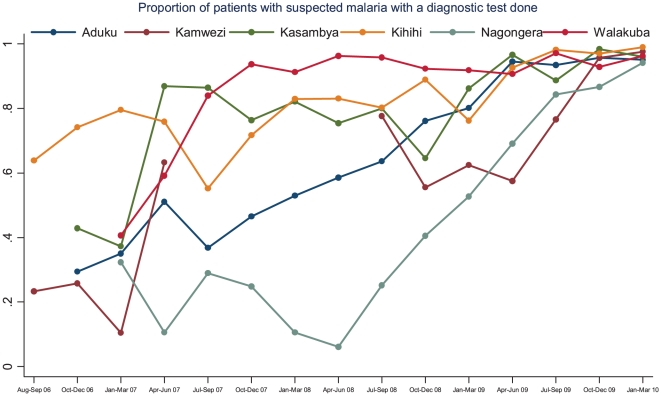
Proportion of patients with suspected malaria with a diagnostic test done by quarter and stratified by site.

The proportion of patients with suspected malaria who underwent diagnostic testing increased from 39% during the first three months of surveillance (range 28–64% at the six sites) to 97% during the last three months of observation (range 94–99% at the six sites) resulting in an absolute increase of 58% (95% CI 57–59%, p<0.001). Temporal changes in the proportion of patients with suspected malaria undergoing diagnostic testing are presented in Figure 3. Five of the six sites had a significant increase following the JUMP training program, however several of the sites had subsequent periods of decline or minimal improvement in the year following training. Over the last year of the observation period all of the sites showed improvement, reaching at least 94% success in obtaining laboratory testing for patients with suspected malaria.

### Antimalarial treatment practices

**Figure 4:**
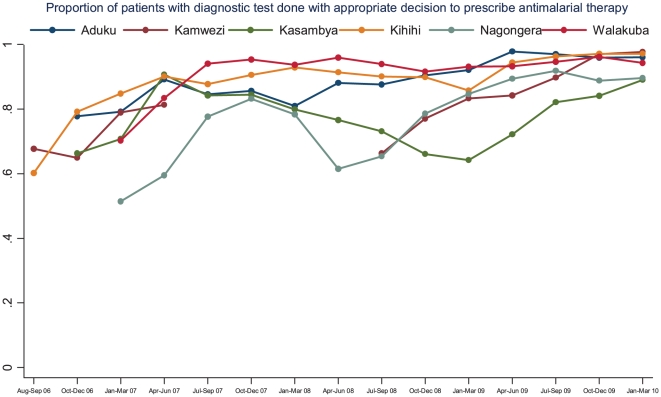
Proportion of patients with diagnostic test done with appropriate decision to prescribe antimalarial therapy by quarter and stratified by site.

The proportion of patients with a diagnostic test done with an appropriate decision to prescribe antimalarial therapy increased from 64% during the first three months of surveillance (range 51–78% at the six sites) to 95% during the last three months of observation (range 89–98% at the six sites) resulting in an absolute increase of 30% (95% CI 29–31%, p<0.001). Temporal changes in the proportion of patients with a diagnostic test done with an appropriate decision to prescribe antimalarial therapy are presented in Figure 4. Most sites showed initial improvement following the JUMP training program followed by a period of decline or relatively little change followed by gradual improvement again during the last year of observation.

Making an appropriate decision to prescribe antimalarial therapy can be divided into prescribing antimalarial therapy in those with a positive diagnostic test and not prescribing antimalarial therapy in those with a negative diagnostic test. Although there was significant improvement in the proportion of those with a positive diagnostic test prescribed antimalarial therapy when comparing the first three months with the last three months (90% vs. 98%, p<0.001), the biggest improvement was in the proportion of patients with a negative diagnostic test not prescribed antimalarial therapy (46% vs. 91%, p<0.001).

**Figure 5:**
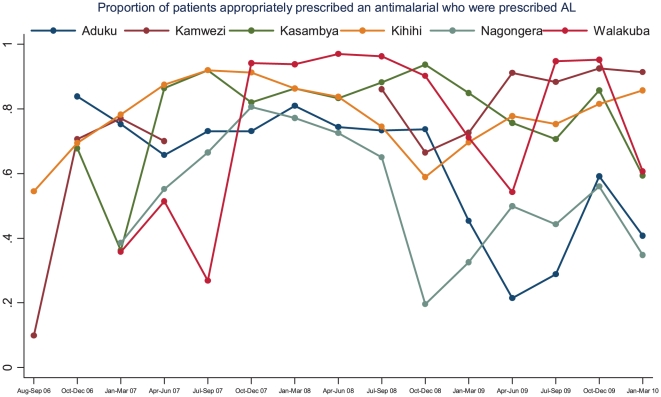
Proportion of patients appropriately prescribed an antimalarial who were prescribed AL by quarter and stratified by site.

The proportion of patients appropriately prescribed antimalarial therapy who were prescribed AL showed a modest increase from 48% during the first three months of surveillance (range 36–84% at the six sites) to 69% during the last three months of observation (range 35–92% at the six sites) resulting in an absolute increase of 20% (95% CI 18–23%, p<0.001). Temporal changes in the proportion of patients appropriately prescribed antimalarial therapy who were prescribed AL are presented in Figure 5. Unlike the other indicators of malaria case management which showed clear improvement over time, trends in AL prescribing practices varied widely over time and between sites. Three sites showed an overall improvement, but did have periods of substantial decline. Two sites showed minimal or no improvement and one site showed a significant decline in the proportion of patients prescribed AL. Of note, less than 2% of patients appropriately prescribed AL were also prescribed another antimalarial drug. Among those with a positive laboratory test result who were not prescribed AL, the most common antimalarials prescribed were quinine (80%) and a combination of chloroquine plus sulfadoxine-pyrimethamine (14%).

## Discussion

During the implementation of a health facility based surveillance system, there were significant improvements in several key steps of malaria case management at the six sentinel sites. The biggest impact was in the proportion of patients with suspected malaria who were referred for diagnostic testing. At the onset of the surveillance system a majority of suspected malaria cases were treated empirically without referral for diagnostic testing. However, utilization of laboratory service greatly improved as 97% of patients with suspected malaria were referred for diagnostic testing during the final three months of evaluation. Treatment practices were also significantly improved in terms of not prescribing antimalarials in patients with negative diagnostic tests, prescribing antimalarials in patients with positive diagnostic tests, and prescribing AL for patients with laboratory confirmed malaria.

The quantitative impact of the surveillance system can be estimated using a simple hypothetical approach comparing observed malaria treatment practices with expected treatment practices assuming no changes in our key indicators after the first three months of observation. Between September 2006 and March 2010, a total of 229,375 cases of suspected malaria were captured by the UMSP surveillance system. Assuming the proportion of patients suspected of malaria with a lab test done and the proportion of patients prescribed antimalarials with positive and negative lab tests would have continued at the same level as the first three months of data collection, the implementation of the surveillance system resulted in 58,678 fewer antimalarial treatments prescribed. In addition, the UMSP almost doubled the number of prescriptions of antimalarials for lab confirmed cases of malaria (32,505 expected vs. 64,366 observed).

The first critical step for improving malaria case management is the referral of patients with suspected malaria for laboratory diagnostic testing. Several studies from Africa have reported less than 50% of patients suspected of having malaria undergo diagnostic testing even when these services are available [10], [11], [12]. The surveillance program described here benefitted from a six-day integrated team-based training course of health care workers conducted shortly after the program was implemented. However, even after this training course, the proportion of suspected malaria cases referred for laboratory testing remained just over 50% [9]. It was not until after over three years of ongoing surveillance and continued supervisory visits that consistent levels of over 90% of suspected malaria cases referred for laboratory testing at all the sites were achieved. Several lessons were learned over the course of these three years. Patience was required as empiric treatment of malaria without diagnostic testing has historically been part of the national policy in most African countries and deeply ingrained in the teaching of health care workers. Indeed, it is only in the last year that the WHO has made a clear recommendation for the laboratory confirmation of diagnosis in all patients suspected of having malaria before treating in situations where diagnostic testing is available [7]. Another important factor was support from the Ugandan Ministry of Health and district focal persons in advocating for the utilization of laboratory services. Feedback and setting targets were also important for encouraging health care workers and building confidence in the value of having a test result for making treatment decisions. Finally, ensuring the laboratories at the sentinel sites were well equipped to handle the large numbers of patients referred for laboratory testing was essential. At most of the sites this primarily involved support for microscopy, which included advocating for adequate laboratory personnel and ensuring adequate supplies needed for making blood smears. At some of the sites, the utilization of RDTs also played a role, especially in areas of unstable transmission intensity where the need for diagnostic testing can fluctuate, and at times overwhelm the capacity for microscopy. However, the role of RDTs was limited by their availability.

The primary objective of the surveillance program was to generate unbiased and precise estimates of the SPR by increasing the utilization of diagnostic testing among cases of suspected malaria. These data are provided to the Ministry of Health and other stakeholders in the form of monthly reports which are also posted on a public website (http://umsp.muucsf.org/). Although evaluations of trends in SPR were beyond the scope of this report, increasing the use of diagnostic testing provided an opportunity to evaluate the impact of the surveillance program on improving antimalarial treatment practices. The use of diagnostic testing may improve patient care in parasite-positive patients, allow for the identification of parasite-negative patients in whom another diagnosis should be sought, reduce the use of unnecessary antimalarials, and provide confirmation of treatment failures. Approximately 1 in 10 patients with a positive diagnostic test were not prescribed antimalarials during the initial period of the surveillance program. This appeared to be due to clinicians making decisions about treatment prior to receiving the diagnostic test results based on informal discussions with clinic staff. Through continuous training and supervisory visits, clinicians were encouraged to wait for the laboratory result before making treatment decisions resulting in a significant reduction in the proportion of parasite-positive patients not prescribed an antimalarial. A much more common problem early in the surveillance program was the practice of prescribing antimalarials in patients with a negative diagnostic test. Indeed, studies across a wide range of epidemiologic setting in Africa have documented that 35–79% of patients with negative diagnostic test result were still prescribed antimalarial drugs [11], [12], [13], [14]. This seemingly irrational treatment practice can be difficult to change as demonstrated by a study in Tanzania where the introduction of RDTs and basic training did not lead to a reduction in overuse of antimalarial drugs [15].

In this surveillance program, the proportion of patients with a diagnostic test done who were appropriately prescribed antimalarial therapy increased after the JUMP training program, however, some sites failed to sustain these improvements or showed declines. Only after three years of the surveillance program were we able to reach levels greater than 90%, although two sites continue to prescribe antimalarials in up to 20% of patients with a negative diagnostic test. Again, promoting rational antimalarial treatment practices took patience, continual feedback to the health care providers, and support from government officials at the Ministry of Health and district level. In the era of ACTs, limiting the unnecessary use of antimalarials becomes a high priority as this will help maintain drug supplies, reduces health system costs [16], [17], and might reduce opportunities for the selection of drug resistant parasites. The surveillance program was less successful in promoting the use of AL in parasite-positive patients. Although there were some modest gains, AL treatment practices varied widely over time and between sites. Although data on the reasons for not prescribing AL were not collected systematically, informal discussions with health care workers suggested that the primary factor responsible was drug stock-outs. As ACTs are being rolled out in large numbers around Africa, the ability to maintain a consistent drug supply has become a major issue and has been cited as a major factor in health care workers choice of antimalarials in Uganda and Kenya [18], [19].

There are several important limitations of this study that should be pointed out. The surveillance program was not implemented as a controlled experiment, therefore causal inferences between the intervention and improvement in indicators of improved malaria case management should be made with caution. Secondly the various components of the surveillance program were not implemented in a systematic fashion. Rather improvements to the program were made over time based on experience, need, and a “trial and error” basis. Indeed, the surveillance program did not establish a pre-specified list of qualitative or quantitative goals in terms of interventions, but rather interventions were developed and implemented as a means of continuously improving indicators of malaria case management. Finally, data collected were limited to the practices of health care workers and did not include exit interviews or follow-up surveys. Therefore it is unknown whether improvements in health care worker performance lead to improved patient outcomes. Several “downstream” factors such as proper dosing, successfully filling prescriptions, adherence to medications, and treatment seeking practices after leaving the clinic are all important for successful malaria case management.

In summary, although the absence of a “control group” limits the ability to make causal inferences, the experience of UMSP provides evidence for the utility of a health facility-based sentinel site malaria surveillance system that produces high quality data in Africa given that adequate resources are available. In addition to improving the capacity to monitor trends in malaria morbidity and measure the impact of control interventions in these selected sites, there is added value by improving malaria case management for large numbers of patients. Indeed, surveillance itself should be considered an intervention and an integral part of any malaria control program. Success of the program did not occur overnight, but rather required patience, flexibility, feedback from heath care workers, and continuous support from the government and funding agency. Although the malaria surveillance program described here has not been expanded beyond the sentinel site health facilities, lessons learned from this program should benefit other initiatives aimed at improving malaria case management in other health care facilities and provides a demonstration project for changing the practices of health care workers in Africa.

## Supporting Information

Appendix S1 **Patient Record Form.**
(PDF)
