# Supplementary material for: Improved Malaria Case Management through the Implementation of a Health Facility-Based Sentinel Site Surveillance System in Uganda
Source: PLoS One. 2011 Jan 19;6(1):e16316. doi: 10.1371/journal.pone.0016316 (PMC3023768; doi:10.1371/journal.pone.0016316)
Supplement: Appendix S1 — Patient Record Form. (PDF) [file pone.0016316.s001.pdf]

Patient Record Form

Clinic________________

Date |___||___|/|___||___|/|___||___|	OPD Number |___||___||___||___|	Patient's Last Name	First Name	New attendance ☐Yes ☐ No
Parish	Village	Age: |___||___| Yrs |___||___| Mos	Sex ☐Male ☐Female	Weight |___||___|.|___|kg

History & Exam findings

Fever or history of fever? ☐ Yes ☐ No | History of cough ≥ 2 weeks? ☐ Yes ☐ No **(BOTH these questions must be completed)**

☐**BS for Malaria** ☐**Pos** ☐ **Neg**<br>**Parasite density:** ________________**(if positive)**<br>☐**RDT for Malaria** ☐**Pos** ☐ **Neg**<br>**Malaria Lab number** |___||___||___||___|	☐**HIV test** ☐**CTRR** ☐ **CTR**<br>**HIV Lab number** |___||___||___||___|	☐**TB exam:**<br>1st smear ☐**Pos** ☐**Neg (today's date)**<br>2nd smear ☐**Pos** ☐**Neg Date:** ___ ___/___ ___/___ ___<br>3rd smear ☐**Pos** ☐**Neg Date:** ___ ___/___ ___/___ ___<br>**TB Lab number** |___||___||___||___|
☐ **Stool ordered** - Results:	☐ **Urinalysis ordered** – Results:	☐**Hb** |___||___|.|___|g/dl<br>☐**VDRL test** ☐**Pos** ☐ **Neg**<br>☐**Other** (test/result)

Diagnosis (Check all that apply)

**Reportable diseaes**	☐ Malaria (not during pregnancy)	☐ Animal and Snake bites	☐ Haemorrhage during pregnancy
☐ Acute flaccid paralysis	☐ Malaria (during pregnancy)	☐ Asthma	☐ High BP during pregnancy
☐ Cholera	☐ Meningitis (Non meningococcal)	☐ Cardiovascular- High BP	☐ Obstructed labour
☐ Dysentery	☐ Onchocerciasis	☐ Cardiovascular- Other	☐ Perinatal conditions in newborns
☐ Guinea worm	☐ Pelvic Inflammatory Disease	☐ Childhood mental disorder	**Miscellaneous Diseases**
☐ Hemorrhagic fever	☐ Pneumonia	☐ Diabetes Mellitus	☐ Death in OPD (no diagnosis)
☐ Measles	☐ Schistosomiasis	☐ Epilepsy	☐ ENT Conditions
☐ Meningitis (Meningococcal)	☐ Sleeping Sickness	☐ GI disorders (non infective)	☐ Eye Conditions
☐ Plague	☐ STI	☐ Injuries- Road traffic accidents	☐ Skin Conditions
☐ Rabies	☐ Tetanus (over 28 days age)	☐ Injuries- Trauma of other origin	☐ Oral Diseases and conditions
☐ Tetanus (0-28 days age)	☐ Typhoid Fever	☐ Malnutrition- low weight for age	☐ Illegible or unclear
☐ Yellow Fever	☐ Urinary Tract Infections (UTI)	☐ Malnutrition- severe	**Other Diagnosis**
**Infectious Disease**	**Tuberculosis**	☐ Mental Illness- Anxiety	
☐ AIDS/HIV	☐ New case – No prior TB treatment	☐ Mental Illness- Depression	
☐ Cough or Cold (no pneumonia)	☐ New case – Previous TB treatment	☐ Mental Illness- Mania	
☐ Diarrhea- Acute	☐ Known TB Case – Med Refill	☐ Mental Illness- Schizophrenia	
☐ Diarrhea- Persistent	**Non Infectious Diseases**	☐ Mental Illness- Other	
☐ Intestinal worms	☐ Alcohol and drug abuse	**Maternal and Perinatal Diseases**	
☐ Leprosy	☐ Anaemia	☐ Abortions	

Treatment (Check all that apply)

Drug	Dose	Drug	Dose
Antimalarial		Other Drugs	
☐ Coartem		☐ Aspirin	
☐ Quinine		☐ Cough linctus	
☐ Chloroquine		☐ Diazepam	
☐ Amodiaquine		☐ Dexamethasone	
☐ SP		☐ Diclofenac	
☐ Artesunate		☐ Folic Acid	
☐ Duocotecxin		☐ Gentian violet	
Antimicrobials		☐ Hydrocortisone	
☐ Albendazole		☐ Ibuprofen	
☐ Amoxilicilin		☐ Magnesium	
☐ Chloramphenicol		☐ Multivitamin	
☐ Ciprofloxacin		☐ Nystatin	
☐ Cloxacillin		☐ Paracetamol	
☐ Cotrimoxazole		☐ Phenytoin	
☐ Doxycycline		☐ Piriton	
☐ Erythromycin		☐ Salbutamol	
☐ Gentamicin		☐ Vit. B group	
☐ Mebendazole		Other	
☐ Metronidazole		Other	
☐ PPF		Other	
☐ Tetracycline		Other	
☐ X-pen		Other	

**Referrals and additional notes**		TB Drug Regimen (Check if prescribed)
☐Admitted to ward	Notes	☐ **Initial Phase**
☐Referred to HIV care		☐RHZE ☐RHZES ☐RHZ
☐Referred for TB care		☐ **Continuation Phase**
☐Referred for other services		☐HE ☐RHE ☐RH

Full Name Signature
